# Supplementary figures and images for: Recruitment of Ahsa1 to Hsp90 is regulated by a conserved peptide that inhibits ATPase stimulation
Source: EMBO Rep. 2024 Jun 27;25(8):20. doi: 10.1038/s44319-024-00193-8 (PMC11316058; doi:10.1038/s44319-024-00193-8)

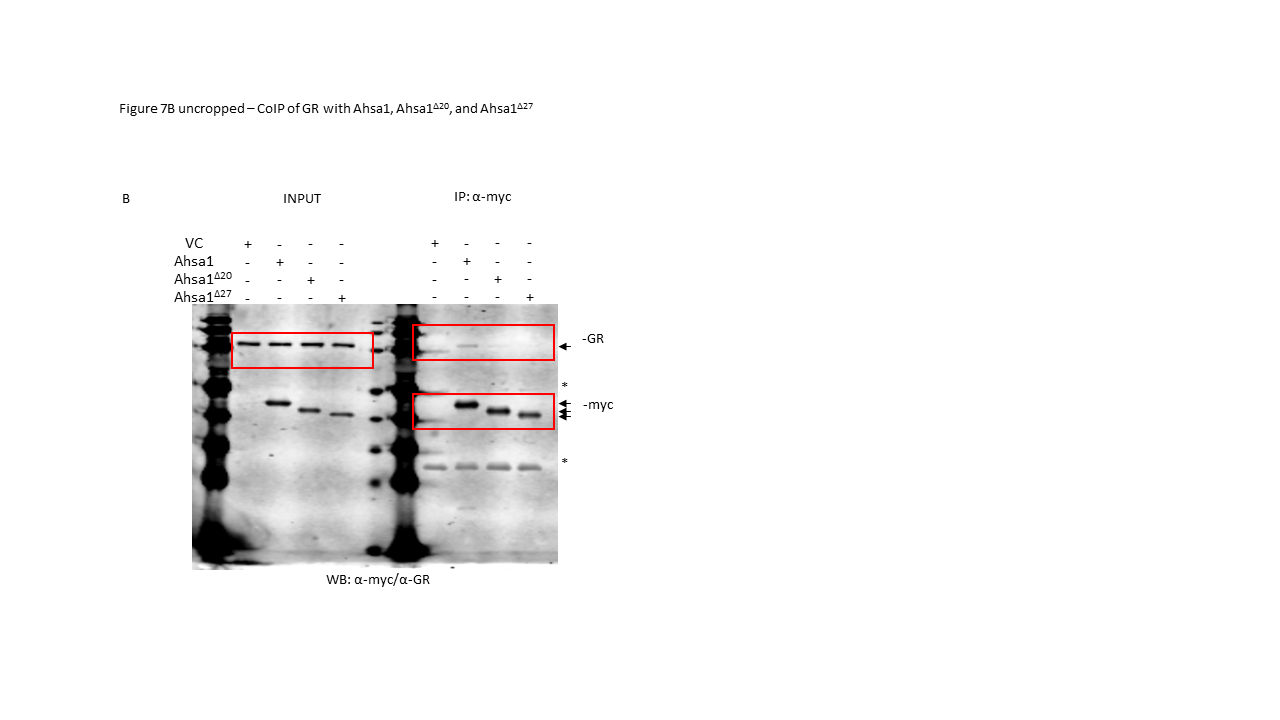

Supplement: Supplementary file 7 — Source data Fig. 7 [file 44319_2024_193_MOESM7_ESM.zip › Figure 7_B.PNG]

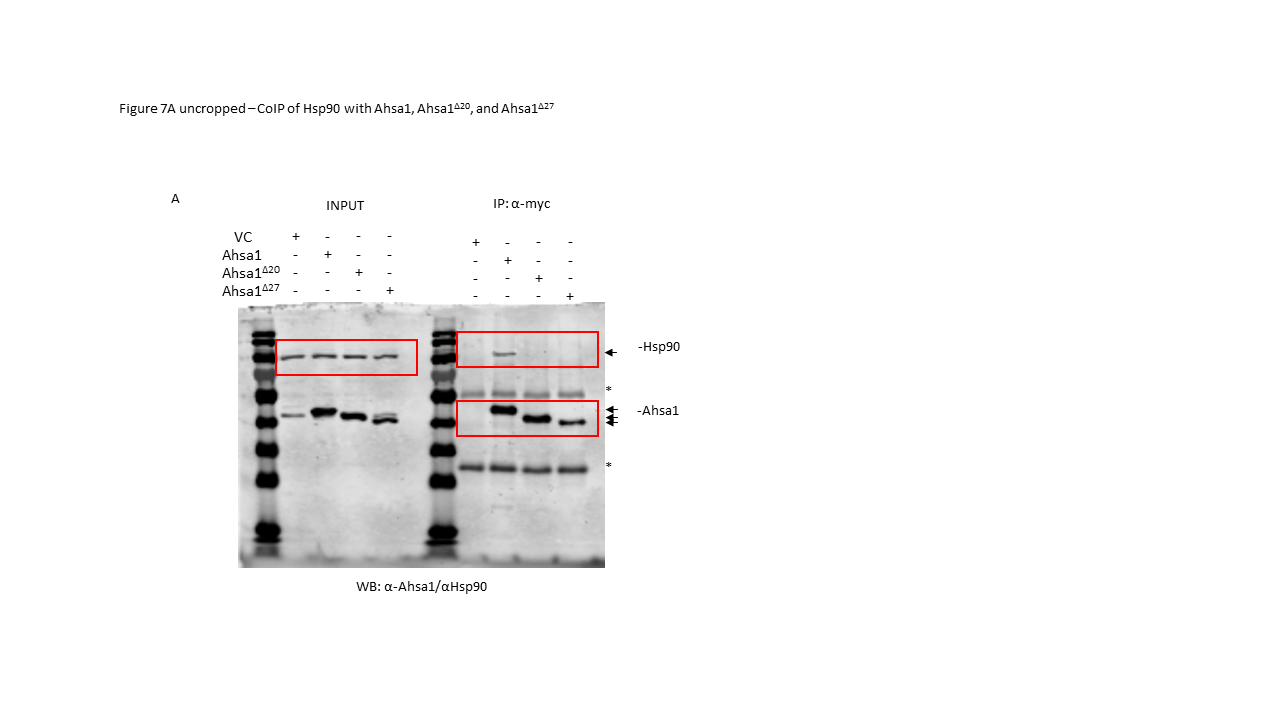

Supplement: Supplementary file 7 — Source data Fig. 7 [file 44319_2024_193_MOESM7_ESM.zip › Figure 7_A.PNG]

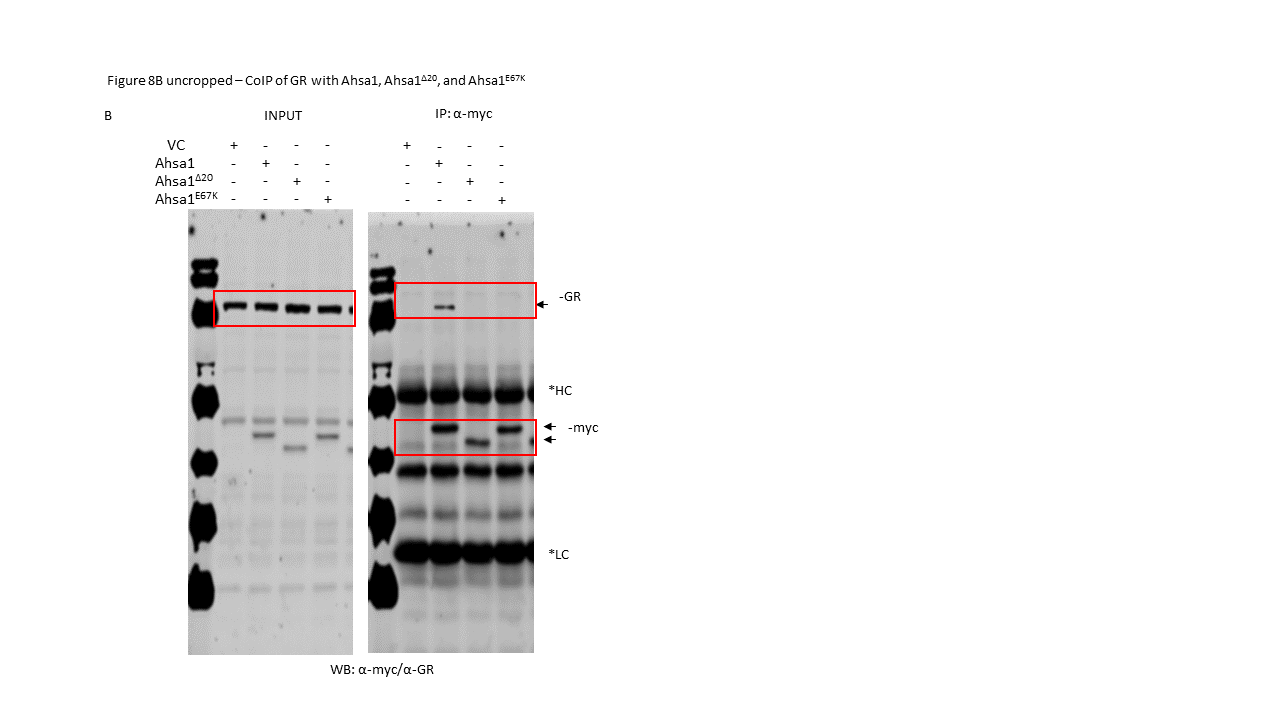

Supplement: Supplementary file 8 — Source data Fig. 8 [file 44319_2024_193_MOESM8_ESM.zip › Figure 8_B.PNG]

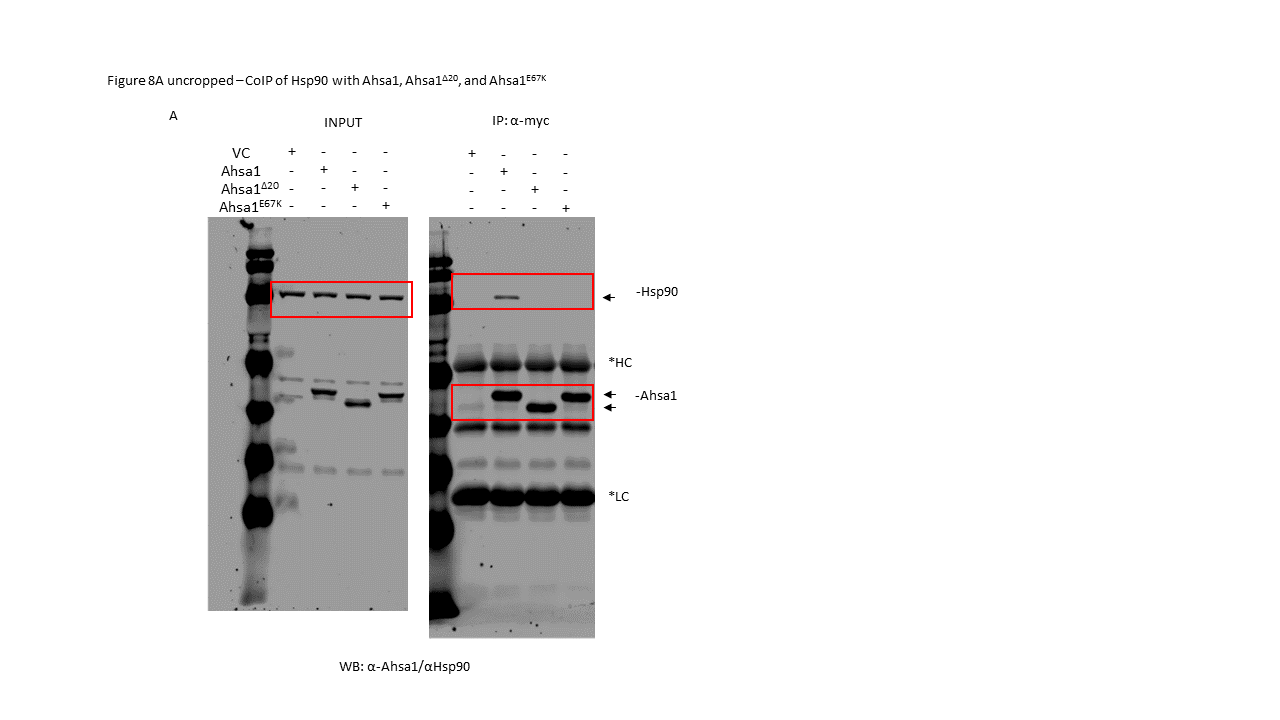

Supplement: Supplementary file 8 — Source data Fig. 8 [file 44319_2024_193_MOESM8_ESM.zip › Figure 8_A.PNG]
