# Supplementary material for: Recruitment of Ahsa1 to Hsp90 is regulated by a conserved peptide that inhibits ATPase stimulation
Source: EMBO Rep. 2024 Jun 27;25(8):20. doi: 10.1038/s44319-024-00193-8 (PMC11316058; doi:10.1038/s44319-024-00193-8)
Supplement: Supplementary file 10 — Expanded View Figures [file 44319_2024_193_MOESM10_ESM.pdf]

Expanded View Figures

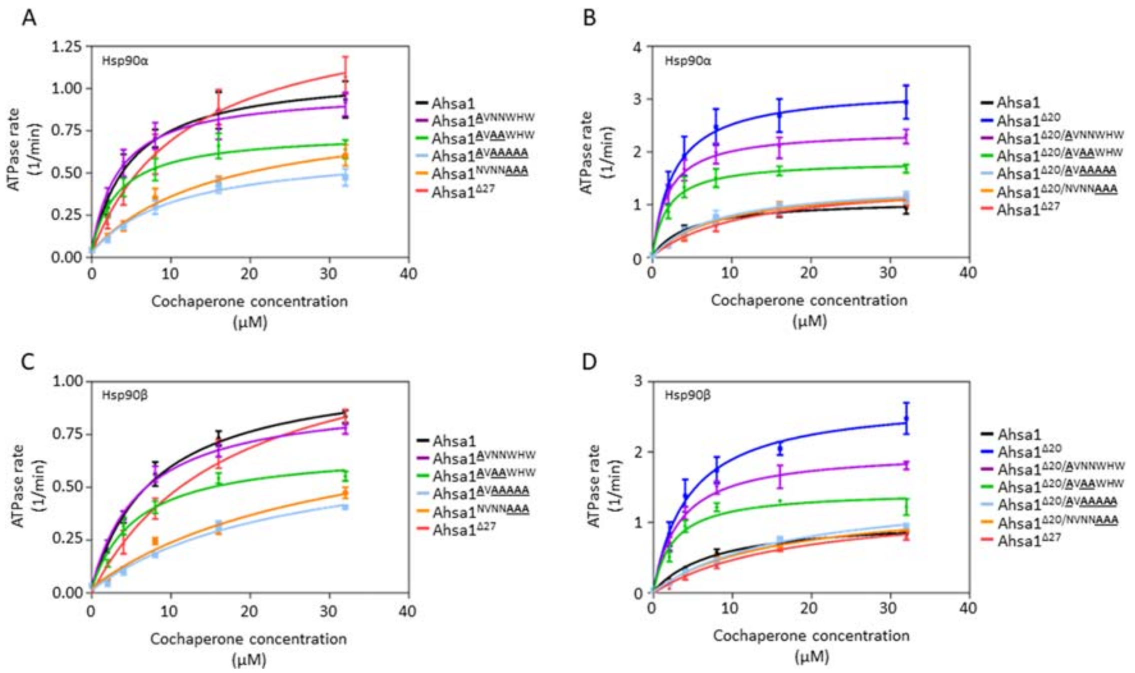

Figure EV1. Point mutations in the NxNNWHW motif impair ATPase stimulation by Ahsa1.

(**A**) Stimulation of Hsp90α ATPase activity by increasing concentrations of full-length Ahsa1 (black), Ahsa1^{AVNNWHW} (purple), Ahsa1^{AVAAWHW} (green), Ahsa1^{NVNNAAA} (orange), Ahsa1^{AVAAAAA} (light blue), and Ahsa1$^{\Delta 27}$ (red). (**B**) Stimulation of Hsp90α ATPase activity by increasing concentrations of full-length Ahsa1 (black), Ahsa1$^{\Delta 20}$ (blue), Ahsa1$^{\Delta 20/AVNNWHW}$ (purple), Ahsa1$^{\Delta 20/AVAAWHW}$ (green), Ahsa1$^{\Delta 20/NVNNAAA}$ (orange), Ahsa1$^{\Delta 20/AVAAAAA}$ (light blue), and Ahsa1$^{\Delta 27}$ (red). (**C**) Stimulation of Hsp90β ATPase activity by increasing concentrations of full-length Ahsa1 (black), Ahsa1^{AVNNWHW} (purple), Ahsa1^{AVAAWHW} (green), Ahsa1^{NVNNAAA} (orange), Ahsa1^{AVAAAAA} (light blue), and Ahsa1$^{\Delta 27}$ (red). (**D**) Stimulation of Hsp90β ATPase activity by increasing concentrations of full-length Ahsa1 (black), Ahsa1$^{\Delta 20}$ (blue), Ahsa1$^{\Delta 20/AVNNWHW}$ (purple), Ahsa1$^{\Delta 20/AVAAWHW}$ (green), Ahsa1$^{\Delta 20/NVNNAAA}$ (orange), Ahsa1$^{\Delta 20/AVAAAAA}$ (light blue), and Ahsa1$^{\Delta 27}$ (red). Reactions contained 1 μM Hsp90α or Hsp90β and indicated concentration of cochaperone. Data Information: All data shown in (**A**, **B**) were obtained in the same 384-well experiment three times ($N = 3$). Data for full-length Ahsa1 constructs were plotted in (**A**) and for Ahsa1$^{\Delta 20}$ constructs were plotted in (**B**). In (**A**, **B**), data for each concentration of cochaperone are presented as mean +/− SEM of 3 independent experiments ($N = 3$; each N is one experiment carried out with technical triplicates as described in "Methods"). All data shown in (**C**, **D**) were obtained in the same 384-well experiment four times ($N = 4$). Data for full-length Ahsa1 constructs were plotted in C and for Ahsa1$^{\Delta 20}$ constructs were plotted in (**D**). (**C**, **D**) Data for each concentration of cochaperone are presented as mean +/− SEM of 4 independent experiments ($N = 4$; each N is one experiment carried out with technical triplicates as described in "Methods"). Source data are available online for this figure.

© The Author(s)

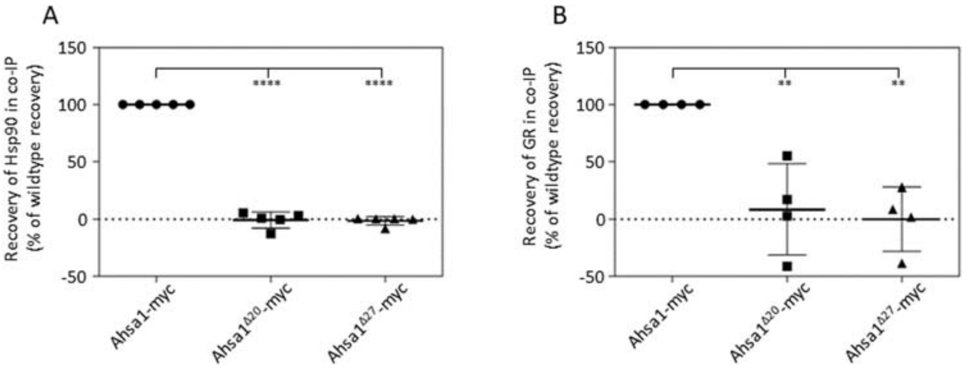

Figure EV2. The ICD is required for stable interaction with Hsp90 and GR.

Band intensity of five replicate experiments was measured by densitometry. After background subtraction, intensities of Hsp90 (**A**) and GR (**B**) were normalized against the amount of Ahsa1 recovered and expressed as a percentage of each protein with wildtype Ahsa1. Data Information: In (**A**), data from five individual experiments are plotted as individual points as well as the mean +/− SD ($N = 5$). In (**B**), data from four individual experiments are plotted as individual points as well as the mean +/− SD ($N = 4$). Statistical significance was calculated for (**A**, **B**) using a Dunnett's multiple comparison's test (**$P \leq 0.01$; ****$P \leq 0.0001$). Source data are available online for this figure.

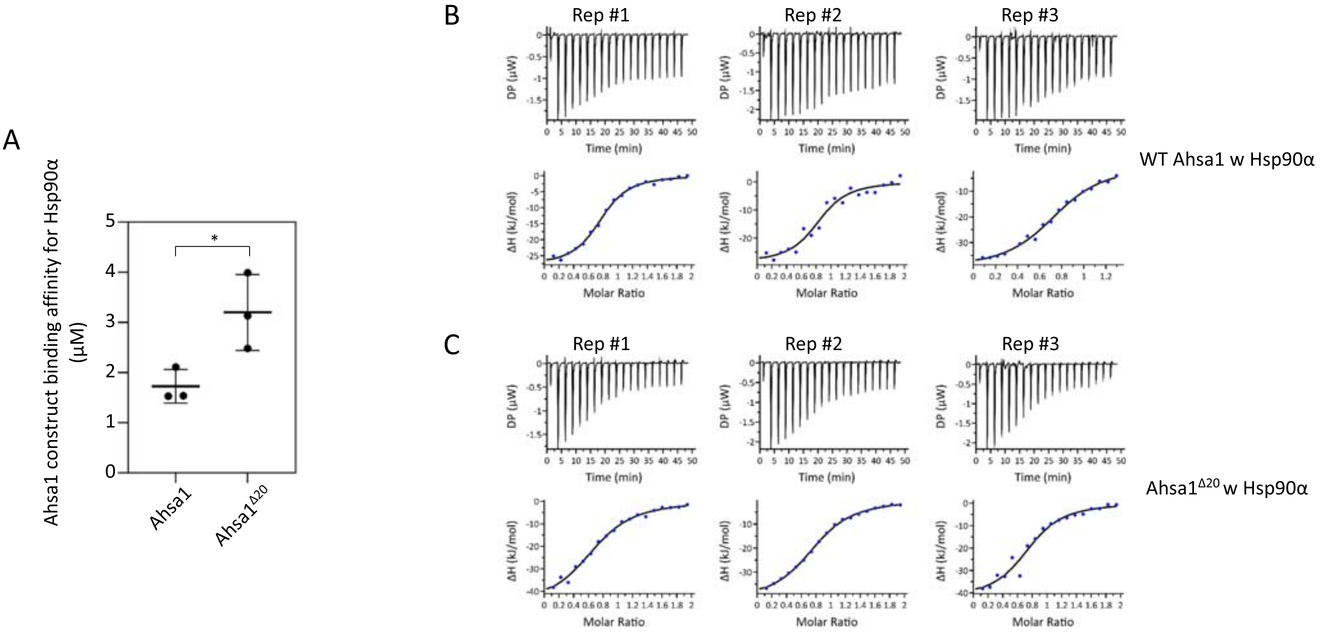

D

Experiment	Temperature (°C)	[Syr] (M)	[Cell] (M)	N (sites)	KD (M)	KD Error (M)	ΔH (kJ/mol)	ΔG (kJ/mol)	-TΔS (kJ/mol)
30uM Hsp90-α-300uM hAhsa1-WT	25.1	3.00E-04	3.00E-05	0.761	1.53E-06	2.58E-07	-28.3	-33.2	-4.93
30uM Hsp90-α-300uM hAhsa1-WT	25.1	3.00E-04	3.00E-05	0.829	1.54E-06	9.08E-07	-29	-33.2	-4.24
30uM Hsp90-α-300uM hAhsa1-WT	25.1	2.00E-04	3.00E-05	0.778	2.11E-06	5.51E-07	-40.4	-32.4	7.98
					1.73E-06	5.72E-07			
30uM Hsp90-α-300uM hAhsa1-**Δ**20	25.1	3.00E-04	3.00E-05	0.716	3.99E-06	1.12E-06	-46.6	-30.8	15.8
30uM Hsp90-α-300uM hAhsa1-**Δ**20	25.1	3.00E-04	3.00E-05	0.802	3.13E-06	3.20E-07	-42.2	-31.4	10.7
30uM Hsp90-α-300uM hAhsa1-**Δ**20	25.1	3.00E-04	3.00E-05	0.766	2.48E-06	1.32E-06	-42.7	-32	10.7
					3.20E-06	9.20E-07			

Figure EV3. Affinity of Ahsa1 and Ahsa$^{\Delta20}$ for Hsp90α.

(**A**) Binding affinity of wildtype Ahsa1 and Ahsa1$^{\Delta20}$ for Hsp90α are shown in the scatter plot. $N = 3$. (**B**, **C**) The final ITC figures are generated with MicroCal PEAQ-ITC analysis software. The upper panels show the baseline-subtracted, singular-value-decomposition (SVD)-corrected thermograms and the bottom panels show the binding isotherms with fitting curves of Hsp90-α titration by hAhsa1-WT (**B**) or Ahsa1$^{\Delta20}$ (**C**), respectively. (**D**) Details of the thermodynamic parameters: (i.e. n stoichiometric ratio of ligand to protein, K_D dissociation constant; ΔH, ΔS and ΔG indicate changes in enthalpy, entropy, and Gibbs free energy, respectively). Data Information: In (**A**), calculated affinities for three experiments are shown as individual points as well as the mean +/− SD ($N = 3$). Statistical significance was calculated using an unpaired t test ($^*P \leq 0.05$). Source data are available online for this figure.

 © The Author(s)

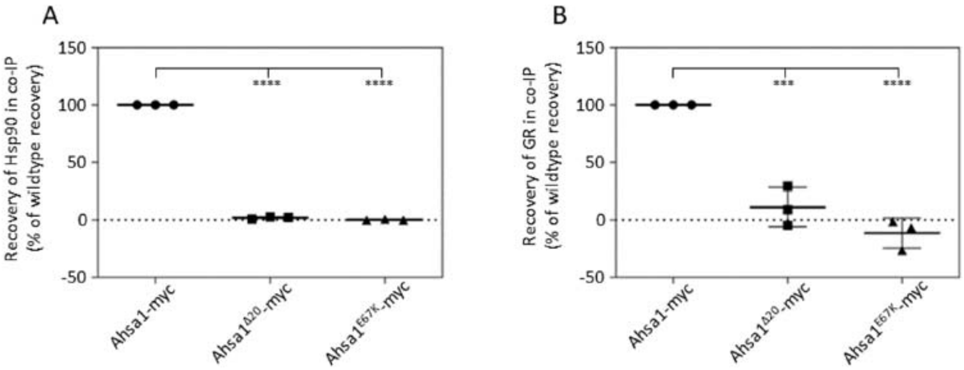

Figure EV4. Complex formation between Ahsa1 and GR requires Ahsa1 interaction with Hsp90.

Band intensity of three replicate experiments was measured by densitometry. After background subtraction, intensities of Hsp90 (**A**) and GR (**B**) were normalized against the amount of Ahsa1 recovered and expressed as a percentage of each protein with wildtype Ahsa1. Data Information: In (**A, B**), data from three individual experiments are plotted as individual points as well as the mean +/− SD ($N = 3$). Statistical significance was calculated for (**A**, **B**) using a Dunnett's multiple comparison's test (***$P \leq 0.001$; ****$P \leq 0.0001$). Source data are available online for this figure.
